# Experimental realization of Bloch oscillations in a parity-time synthetic silicon photonic lattice

**DOI:** 10.1038/ncomms11319

**Published:** 2016-04-20

**Authors:** Ye-Long Xu, William S. Fegadolli, Lin Gan, Ming-Hui Lu, Xiao-Ping Liu, Zhi-Yuan Li, Axel Scherer, Yan-Feng Chen

**Affiliations:** 1 National Laboratory of Solid State Microstructures, Nanjing University, Nanjing, Jiangsu 210093, China; 2 Department of Physics and Kavli Nanoscience Institute, California Institute of Technology, Pasadena, California 91125, USA; 3 Laboratory of Optical Physics, Institute of Physics, Chinese Academy of Sciences, Beijing 100190, China; 4 Collaborative Innovation Center of Advanced Microstructures, Nanjing University, Nanjing 210093, China

## Abstract

As an important electron transportation phenomenon, Bloch oscillations have been extensively studied in condensed matter. Due to the similarity in wave properties between electrons and other quantum particles, Bloch oscillations have been observed in atom lattices, photonic lattices, and so on. One of the many distinct advantages for choosing these systems over the regular electronic systems is the versatility in engineering artificial potentials. Here by utilizing dissipative elements in a CMOS-compatible photonic platform to create a periodic complex potential and by exploiting the emerging concept of parity-time synthetic photonics, we experimentally realize spatial Bloch oscillations in a non-Hermitian photonic system on a chip level. Our demonstration may have significant impact in the field of quantum simulation by following the recent trend of moving complicated table-top quantum optics experiments onto the fully integrated CMOS-compatible silicon platform.

## Abstract (web summary)

Bloch oscillations are a fundamental electron transport phenomenon in condensed matter. Here, the authors experimentally realize spatial Bloch oscillations in a non-Hermitian integrated photonic system using CMOS compatible fabrication processes, contributing to improving the understanding of non-Hermitian transport phenomena.

## Graphical abstract

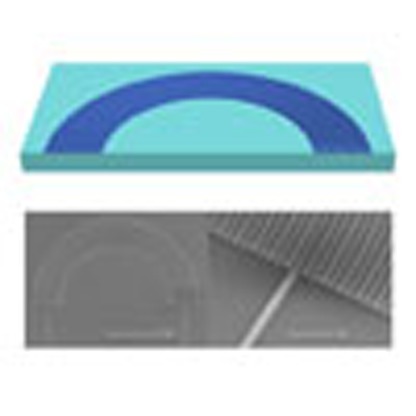

Bloch oscillations (BO) effect is a fundamental electron transport phenomenon in condensed matter, which is characterized as the coherent oscillatory motion of electron in a periodic potential driven by an external DC electric field^1^. The electron is accelerated by the electric field, then reflected when it reaches a momentum satisfying the Bragg reflection condition in the periodic potential and, subsequently, decelerated to the initial state to form a BO cycle. However, because of dephasing effects, it is difficult to observe BO in natural crystals. Owing to the development of semiconductor technology, BO have been experimentally observed in semiconductor superlattices with periodically arranged layers of different semiconductor materials^2^. Due to the equivalence between the Schrödinger equation in quantum mechanics and the classical wave equation, BO can be also observed for matter waves^3^,^4^,^5^,^6^, optical waves^7^,^8^,^9^,^10^,^11^,^12^,^13^, acoustic waves^14^ and surface plasmon polariton waves^15^. In fact, studying BO and related wave transportation phenomena in some of these waveforms can be very advantageous compared to electrons. For example, coherent optical sources with much longer coherent length are readily available, so the dephasing effect would not become a hurdle. Moreover, advanced nanofabrication technologies allow for the creation of very complicated optical potentials, which has already become a major driving force behind the rapid development of the latest nano-photonic technologies. It is worth pointing out that almost all the experimental studies involving BO are primarily focused on the wave dynamics in Hermitian systems with only real-valued potentials. Recently, a particular class of non-Hermitian photonic systems with parity-time (PT) symmetry has drawn great research interest^16^,^17^,^18^,^19^,^20^,^21^,^22^,^23^,^24^,^25^. It is shown that such systems can possess a spontaneous symmetry-breaking phase transition with an eigen energy transition from real spectra into complex spectra. The phase transition point shows all the characteristics of an exceptional point, that is, having non-Hermitian degeneracy, where the real and imaginary parts of eigenvalues are identical. Different from conventional Hermitian degeneracy, not only the eigenvalues but also the eigenvectors coalesce at an exceptional point, giving rise to abundant intriguing physical phenomena. These include unidirectional BO^26^, unidirectional invisibility^27^,^28^,^29^,^30^ and unidirectional transmission^31^. In addition, the exceptional point singularity also has broadened the scope of metamaterials^32^,^33^,^34^.

Here we report an experimental study of photonic BO and related wave dynamics in Hermitian and non-Hermitian systems realized with complementary metal-oxide-semiconductor (CMOS)-compatible fabrication processes on a silicon-on-insulator (SOI) platform. A real-valued periodic potential in a Hermitian photonic lattice in conjunction with a linear gradient potential perturbation acting as a constant acceleration force is created by bending an array of identical SOI waveguides, while a complex PT synthetic potential in a non-Hermitian photonic lattice is realized with the same lattice but with an additional optical loss modulation layer, that is, a chrome (Cr) layer on top of every other bent silicon waveguide. Compared with all other platforms for studying BO and its related classic or quantum wave dynamics^2^,^3^,^4^,^5^,^6^,^7^,^8^,^9^,^10^,^11^,^12^,^13^,^14^,^15^, our integrated platform demonstrated here shows great advantages in emerging applications requiring ultimate scalability and stability, for instance, quantum optics on a chip^35^,^36^,^37^,^38^,^39^,^40^. In addition, by using scanning near-field optical microscope (SNOM), we are able to directly visualize the wave dynamics responsible for the BO continuously for both the Hermitian and non-Hermitian photonic lattice in spatial domain, which is another key advantage over the large-scale discretized PT-symmetric optical networks^30^, where the wave dynamics is discrete in temporal domain and all waves are needed to be multiplexed with actively controlled optical switching components to construct the BO in temporal domain. Our spatial wave dynamics recorded by the SNOM reveal a prominent secondary emission around the BO recovery point for the non-Hermitian system in contrast to the classical picture of BO in a Hermitian system. This feature in such a PT synthetic photonic lattice is related to the spontaneous PT symmetry breaking.

## Results

### BO in a Hermitian photonic lattice

**Figure 1:**
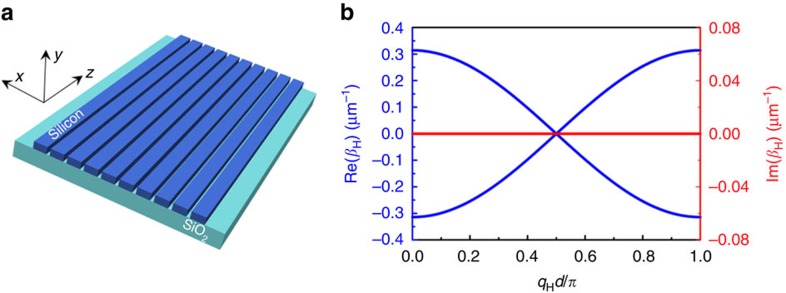
Structure and band diagram of the Hermitian straight photonic lattice. (**a**) Schematic of the photonic lattice consisting of an array of straight SOI waveguides. All SOI waveguides are stripe waveguides 400 nm wide and 220 nm thick and the array period is 500 nm. The individual SOI waveguide has a propagation constant *k=n*_eff_*k*_0_=2.114*k*_0_, while the adjacent SOI waveguides have a coupling coefficient *κ*=0.157 μm^−1^ in this lattice configuration. (**b**) The band diagram for this photonic lattice. Blue and red curves represent the real and imaginary parts of the longitudinal propagation constants, respectively.

**Figure 2:**
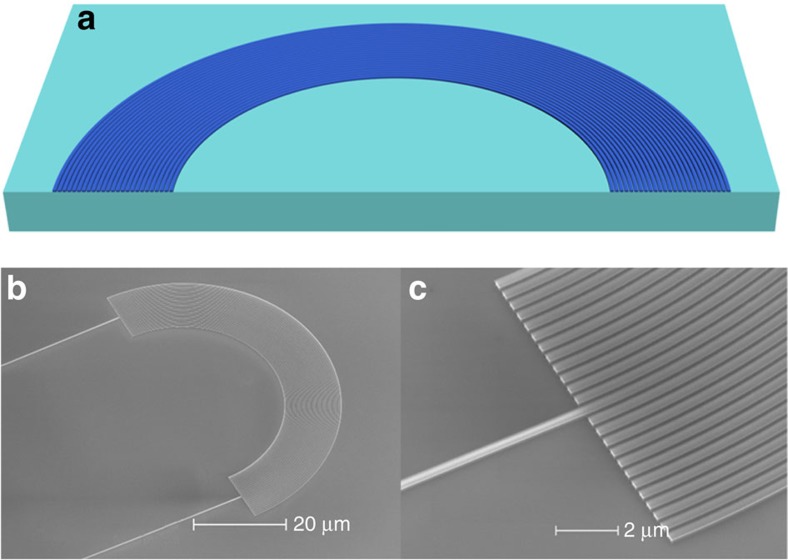
Structure of the Hermitian bent photonic lattice. (**a**) Schematic of the Hermitian bent photonic lattice. The bending radius of the silicon waveguides ranges from *R*=22.7 to 34.7 μm. (**b**) SEM picture of the fabricated device where the input/output waveguide is located at the centre of the photonic lattice and has a bending radius *R*=28.7 μm. (**c**) Zoom-in SEM view of the device. (**b**,**c**) Scale bar, 20 μm and 2 μm, respectively.

As depicted in Fig. 1a, our Hermitian photonic lattice consists of an array of straight equally spaced identical SOI stripe waveguides, which support only a fundamental transverse electric mode at the wavelength of 1,550 nm. In the tight-binding approximation, the propagation of optical wave in this Hermitian photonic lattice is described by coupled-mode equations


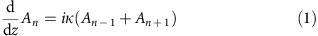


where *Α*_*n*_ is the propagating amplitude in the *n*th waveguide, and *κ* is the coupling coefficient between adjacent waveguides. The corresponding dispersion relation is determined (see Methods) to be


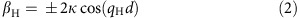


where *d* is the centre distance between adjacent waveguides and *β*_H_ and *q*_H_ are the longitudinal propagation constant and the transverse Bloch momentum of the Hermitian photonic lattice, respectively. A plot of this dispersion relation or band diagram is shown in Fig. 1b, where the blue and red curves represent the real and imaginary parts of the band, respectively. As expected, in this Hermitian photonic lattice the imaginary part of the band is always equal to zero regardless of the Bloch momentum. To obtain Hermitian BO, the Hermitian photonic lattice is bent to modify its spatial distribution of optical potential, as sketched in Fig. 2a. The curvature is seen as inertial force acting on the optical wave, and the linear position dependence of curvature in the radial direction thus can result in a linear potential gradient, which ultimately gives rise to an equivalent DC field required for BO. The corresponding dynamic evolution of this Hermitian bent photonic lattice becomes


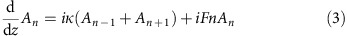


where *Α*_*n*_ is the mode envelop for the *n*th waveguide, and 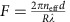 is the equivalent DC field corresponding to the equivalent index gradient in this particular kind of photonic lattice systems (Supplementary Note 1). In the presence of this DC field, the eigenmode can be represented as 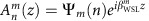, where 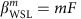, *m*=0, ±1, ±2, … is Wannier-Stark ladder and Ψ_*m*_(*n*) are the corresponding Wannier-Stark eigenstates in the Wannier representation (Supplementary Note 2). We can see that the equivalent DC field *F* acts as a constant Wannier-Stark ladder spacing, which induces field recovery with Bloch period 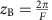, and Wannier-Stark eigenstate is a localized optical mode caused by total internal reflection on the low-index side and Bragg reflection on the opposite high-index side.

**Figure 3:**
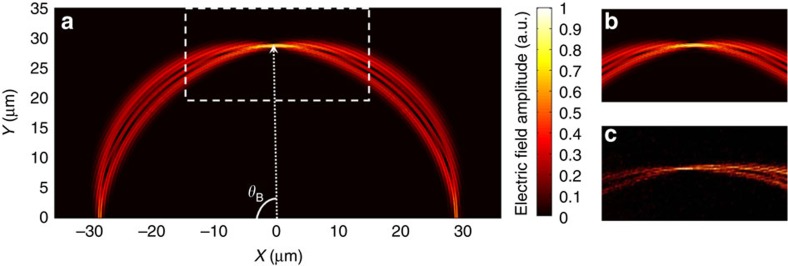
Simulation and experimental observation of the Hermitian BO. (**a**) Simulated electric field amplitude distribution when a single waveguide, that is, the input waveguide is excited. (**b**) Zoom-in view of the simulated electric field amplitude distribution in an area of 30 μm × 19 μm in the vicinity of the first Bloch recovery point. (**c**) Experimentally recorded electric field distribution for the same area using a scanning near-field optical microscope.

To explore the wave dynamics of this photonic lattice, a single waveguide is selectively excited at the input plane. The corresponding evolution of the mode in this waveguide resembles a breathing mode^9^,^10^. To verify this characteristic, we simulate this photonic lattice by means of 3D finite-difference time-domain (FDTD) simulations where the electric field distribution is shown in Fig. 3a. The optical wave initially couples from the centre waveguide into the parallel waveguides towards the transverse and forward direction, and then after propagating pass a certain curvature point (approximately half of Bloch Period) it starts to converge back to a recovering point, which forms a Bloch period 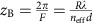, thus finishing a breathing cycle or a BO cycle. The Bloch period can also be conveniently expressed as an in-plane polar angle 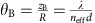. As seen from Fig. 3a, the analytical calculated value *θ*_B_≈84° agrees well with the simulation. In experiment, this Hermitian bent photonic lattice is fabricated using CMOS-compatible nanofabrication processes (see Methods). A scanning electron micrograph of the fabricated device is shown in Fig. 2b,c. The input waveguide used as the single waveguide excitation is located at the centre of the bent photonic lattice. The electric field distribution of the optical wave recorded by a SNOM (see Methods) in the area of 30 μm × 15 μm within the vicinity of the first BO recovery point is shown in Fig. 3c. As would be expected for a Hermitian BO system, the experimental result indicates that the dynamics of the optical wave has a breathing motion and possesses a very distinct recovery point. This experimental observation in fact agrees very well with the simulated electric field distribution shown in Fig. 3b.

### BO in a non-Hermitian photonic lattice

**Figure 4:**
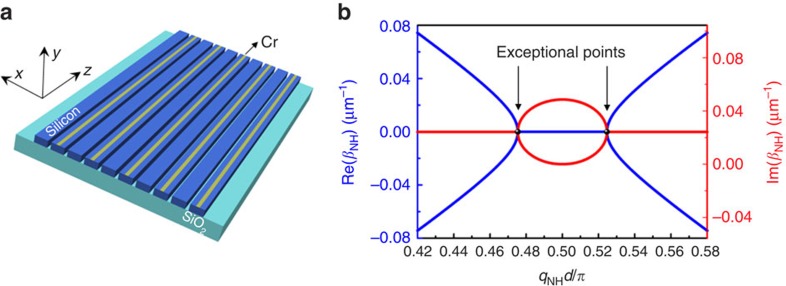
Structure and band structure of the non-Hermitian straight photonic lattice. (**a**) Schematic of the non-Hermitian photonic lattice consisting of a dissipative PT silicon waveguide array. This non-Hermitian photonic lattice has the exact same geometric design as the Hermitian photonic lattice, except for the additional 100-nm-wide and 4-nm-thick chrome (Cr) layer on top of every other silicon waveguide to introduce periodic optical loss modulation with a filed attenuation coefficient *γ*=0.00486 μm^−1^. (**b**) The band diagram of such non-Hermitian photonic lattice. Blue and red curves represent the real and imaginary parts of the longitudinal propagation constants, respectively. The exceptional singularity points are indicated and highlighted as the black points.

PT synthetic photonic structures exhibit intriguing and unusual wave dynamics due to the presence of symmetry breaking, resulting in phase transition from a Hermitian system to a non-Hermitian system. To obtain strict photonic PT symmetry, it requires precise engineering of the spatial distribution of complex optical potential by using optical gain and loss media. However, gain media is not straightforward to be incorporated into the CMOS-compatible fabrication process; therefore, instead of gain, a class of dissipative PT symmetry systems^19^,^29^ with an average background loss have been widely adopted in the literature to study PT-related physics. Here the dissipative element consists of an array of lossy metal stripes deposited on top of every other silicon waveguide of the photonic lattice, as shown in Fig. 4a. By referencing to the background loss, the optical wave in silicon waveguides without (with) metal strips experiences an equivalent gain (loss). The dynamic evolution of the straight photonic lattice, initially Hermitian, is drastically altered by the presence of the synthetic dissipative PT symmetric potential. This change is reflected in the coupled-mode equations governing the dynamics of the mode amplitude *Α*_*n*′_:


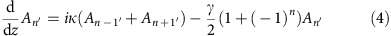


where there exists an alternating mode attenuation (0↔*γ*/2) in adjacent waveguides in addition to the mode coupling *κ* between them. The dispersion relation governing the wave dynamics of this PT synthetic photonic lattice is determined (see Methods) as


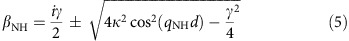


which is plotted in Fig. 4b. This class of dissipative PT symmetric systems possesses two exceptional point singularities at 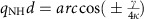 as highlighted by the black points, where both the real and imaginary parts of the band diagram are degenerate. Between these two exceptional point singularities, the state of the system falls into a symmetry-broken phase with a coalesced real part of the band but bifurcated imaginary part. Since for any *γ*>0 there will also be complex eigenvalues near the Brillouin Zone edge (*q*_NH_=*π*/(2*d*)), the PT symmetry breaking is spontaneous or in other words threshold-less. However, it is worth noting that the threshold-less PT symmetry breaking in the straight photonic lattice discussed here is local, where PT symmetry breaking only happens for particular k-vectors along the transverse direction^20^, which is strongly related to optical excitation conditions.

**Figure 5:**
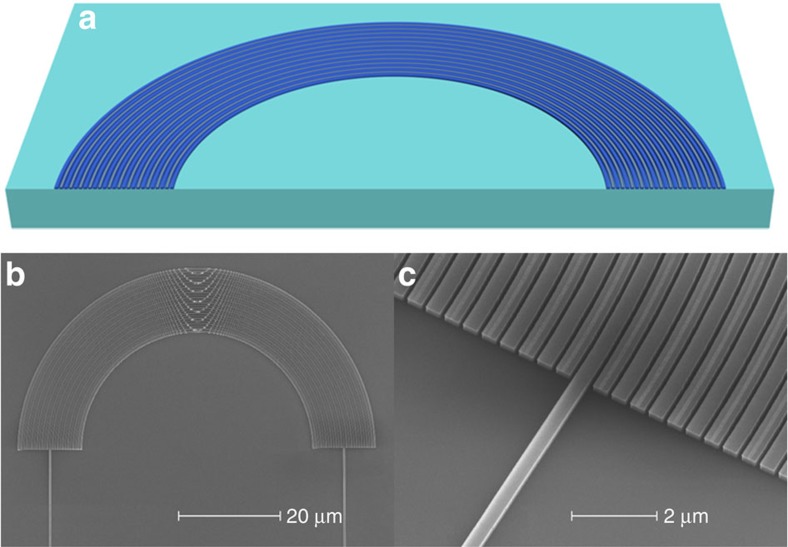
Structure of the non-Hermitian bent photonic lattice. (**a**) Structure of the non-Hermitian bent photonic lattice. This non-Hermitian lattice has the exact same geometrical design as the Hermitian counterpart. (**b**) SEM picture of the fabricated device where the input/output waveguide is located at the centre of the photonic lattice. (**c**) Zoom-in SEM view of the device. The 100-nm-wide and 4-nm-thick Cr layer can be seen on top of every other silicon waveguide. (**b**,**c**) Scale bar, 20 and 2 μm, respectively.

**Figure 6:**
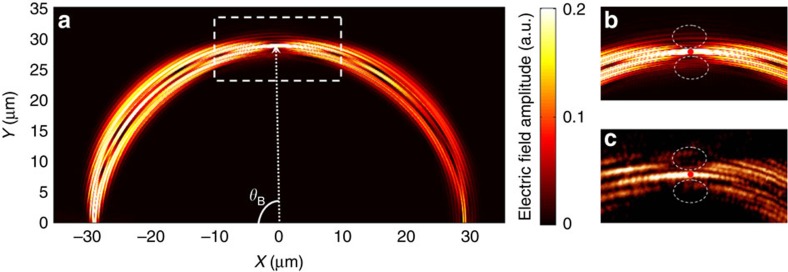
Simulation and experimental observation of the non-Hermitian BO. (**a**) Simulated electric field amplitude distribution when a single waveguide, that is, the input waveguide, is excited. (**b**) Zoom-in view of the simulated electric field amplitude distribution in an area of 20 μm × 10 μm in the vicinity of the first Bloch recovery point. (**c**) Experimentally recorded electric field distribution for the same area using a scanning near-field optical microscope. The white dotted circles mark the secondary emission region, while red dots mark the recovery point in **b**,**c**.

Similarly to the realization of BO in the Hermitian photonic lattice, a constant DC field is also mimicked by a circular bending of the lattice, as sketched in Fig. 5a. In the presence of this DC field the spectra have two interleaved Wannier-Stark ladders 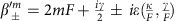, *m*=0, ±1, ±2, …, where 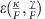 is a complex function of 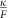 and 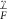 (Supplementary Fig. 1 and Supplementary Note 3). In other words, the eigen energy of a classical BO is split into two in a complex energy plane to reflect the emergence of two localized modes, one with pure optical loss and the other with equivalent optical gain biased by the background loss. The splitting of eigen energy level is a direct outcome of the PT symmetry breaking near the Brillouin boundary. Note that the PT symmetry breaking phenomenon for a particular Bloch mode of the previously described non-Hermitian straight photonic lattice occurs when *γ*>4*κ*cos(*q*_NH_*d*) as shown in Fig. 4b. In this bent configuration the PT symmetry breaking can always be experienced by the optical wave under any excitation condition, because the transverse wave vector in the initial excitation will change under the acceleration force caused by the bending and it will eventually approache the edge of the Brillouin zone, where the threshold-less PT symmetry breaking takes place. To elucidate this, the optical wave dynamics in the non-Hermitian bent photonic lattice is simulated and shown in Fig. 6a. The dynamics of BO in this case still possesses a periodic feature, but with decreasing intensity due to the dissipative background of this system. The BO here, however, is accompanied by a noticeable secondary emission at the BO recovery point as indicated in the zoom-in view of Fig. 6b. In experiment, the designed non-Hermitian bent photonic lattice is fabricated with an additional Cr metal layer on top of the Hermitian bent photonic lattice (see Methods). An example of a fabricated device is shown in Fig. 5b,c, where the waveguide bending radius and the input/output waveguide follow the same design as its Hermitian counterpart. The near-field electric field distribution of the optical wave in this non-Hermitian system is recorded by a SNOM and is shown in Fig. 6c. The overall electric field distribution obtained experimentally near the BO recovery point (indicated as red dots) shows good agreement with the theoretical results in Fig. 6b. Although some of the finest features are not well resolved due to the instrumental limitation, the secondary emission (highlighted in while dotted circles) is clearly visible. Such a emission is originated from the presence of two Wannier-Stark ladders as a result of the spontaneous PT symmetry breaking experienced by the optical wave as it propagates down the photonic lattice with its transverse wave vector evolving to touch the Brillouin zone edge (*q*_NH_=*π*/(2*d*)) under the gradient force *F* as shown in Fig. 4b.

## Discussion

By engineering the spatial distribution of the complex potential for photonic lattices on a SOI photonic platform, we have experimentally demonstrated photonic BO in both a Hermitian and a non-Hermitian system. Apart from the classic oscillatory evolution of the optical wave in Hermitian BO, the dynamics of non-Hermitian BO exhibits an intriguing feature characterized by a secondary emission formed near the BO recovery point. This unique feature is closely related to the spontaneous/threshold-less PT symmetry-breaking phase transition for the BO in our dissipative PT synthetic photonic lattice. The fully integrated non-Hermitian photonic lattice provides a major step forward in expanding the application scope of on-chip photonics in quantum simulation^35^,^36^,^37^,^38^,^39^,^40^,^41^,^42^. In fact, the threshold-less PT symmetry-breaking characteristic in our passive system makes it a perfect candidate for studying the quantum version of non-Hermitian BO as long as the loss modulation is kept low enough to allow for a meaningful high-fidelity quantum measurement, which, however, is difficult to conduct in a gain-loss balanced non-Hermitian system because of the degradation of the initial quantum input state by the inherent quantum noise inside the gain medium. Moreover, thanks to state-of-the-art nano-fabrication technology, which allows for the deliberately designed complex potential distribution to be created, this CMOS-compatible photonic platform with great flexibility opens the door towards the exploration of unprecedented non-Hermitian transport phenomena on a chip^43^,^44^,^45^,^46^,^47^,^48^.

## Methods

### Sample fabrication

The Hermitian photonic lattice was fabricated by means of a single step of electron-beam (e-beam) lithography to define the silicon waveguide layer, which was followed by resist development and inductively coupled plasma etching using a mixture of SF_6_ and C_4_F_8_. The non-Hermitian photonic lattice was fabricated using two steps of aligned e-beam lithography. First, the Cr layer was defined by using a positive e-beam resist and developed, followed by 4 nm of Cr deposition by means of e-beam deposition and liftoff. The silicon waveguides were defined by means of a second step of aligned e-beam lithography using a negative resist, followed by development and finally inductively coupled plasma dry etching using a mixture of etchant aforementioned to define the waveguides. Both photonic lattices went through additional final processing steps before optical testing. These include (1) a photolithographic process to fabricate SU-8 polymer waveguide-based mode converters surrounding the silicon input/output waveguides and (2) dicing and facet polishing.

### Dispersion calculation

Coupled mode equations describing the wave dynamics in both the Hermitian and non-Hermitian photonic lattices were solved to obtain the dispersion spectrum or band diagram. This was done by inserting a plane wave ansatz *Α*_*n*_∝exp(i*β*z−i*nqd*) for the *n*th waveguide's mode amplitude into the coupled mode equation, that is, equations (1) and (4), and by enforcing the non-trivial solutions for *Α*_*n*_. The corresponding transverse Bloch momentum *q*-dependent dispersion *β* in a complex wave-vector domain represents the underlying dispersion spectrum or band diagram.

### Measurement

In measurements, a near-infrared optical wave at the wavelength of 1,550 nm from a tunable external cavity laser (Photonetics, 1,500–1,640 nm) was launched into the SU-8 mode converter through a lensed tapered single-mode fibre. The testing chip and the lensed fibre are bonded onto a glass slide so that the relative position of the fibre to the waveguide is fixing when the testing chip is translated on a moving stage under a SNOM (SNOM-100 Nanonics). The SNOM is equipped with a metal-coated fibre tip with a 50 nm radius to map the intensity distribution. A schematic of our measurement setup is shown in Supplementary Fig. 2 and details of this setup are included in Supplementary Note 4.

## Additional information

**How to cite this article:** Xu, Y.-L. *et al*. Experimental realization of Bloch oscillations in a parity-time synthetic silicon photonic lattice. *Nat. Commun.* 7:11319 doi: 10.1038/ncomms11319 (2016).

## Supplementary Material

Supplementary Information Supplementary Figures 1 & 2, Supplementary Notes 1-4 and Supplementary References.
