# Supplementary material for: Experimental realization of Bloch oscillations in a parity-time synthetic silicon photonic lattice
Source: Nat Commun. 2016 Apr 20;7:11319. doi: 10.1038/ncomms11319 (PMC4842984; doi:10.1038/ncomms11319)
Supplement: Supplementary Information — Supplementary Figures 1 & 2, Supplementary Notes 1-4 and Supplementary References. [file ncomms11319-s1.pdf]

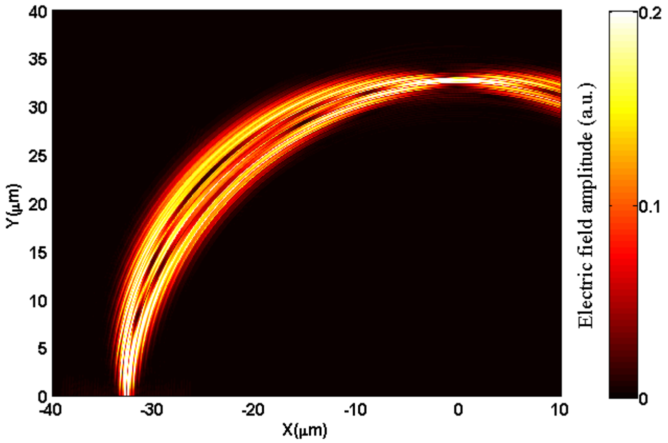

Supplementary Figure 1 Simulated electric field amplitude distribution of the non-Hermitian BO where the input/output waveguide is located at the center of the photonic lattice with a bending radius $R_c = 32.7\mu m$

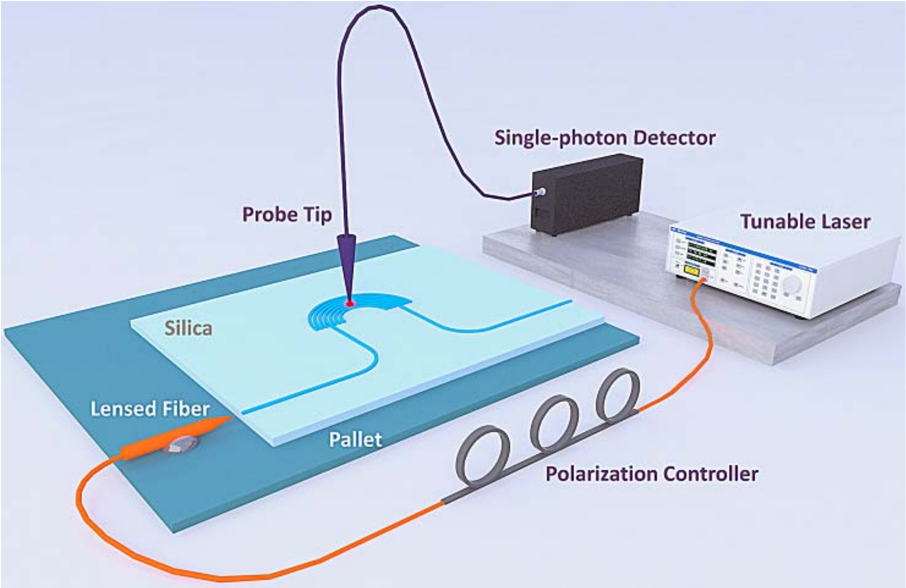

Supplementary Figure 2 Scanning near-field optical microscope (SNOM) measurement setup.

Supplementary Note 1

To determine the effective DC field induced by waveguide curving, conformal transformation can be taken to transform curve waveguides in x, y coordinate system into straight waveguides in u, v coordinate system[1]. Under the transformation, the two dimensional scalar wave equation

$$\left[\frac{\partial^2}{\partial x^2}+\frac{\partial^2}{\partial y^2}+k_0^2 n_{eff}^2\left(x, y\right)\right]E\left(x, y\right)=0 \tag{1}$$

can be expressed as

$$\left[\frac{\partial^2}{\partial u^2}+\frac{\partial^2}{\partial v^2}+\left|\exp\left(\frac{u}{R}\right)\right|^2 k_0^2 n_{eff}'^2\left(u, v\right)\right]E\left(u, v\right)=0 \tag{2}$$

where R is the radius of the curvature, k_0 is the wave number of the light in vacuum, n_{eff} is the effective index. The effective index of the waveguide after transformation is shown as

$$n_{eff}'\left(u, v\right)=\left|\exp\left(\frac{u}{R}\right)\right| n_{eff}\left(u, v\right)\approx\left(1+\frac{u}{R}\right)n_{eff}\left(u, v\right) \tag{3}$$

Then the effective index gradient is given by $\Delta n_{eff} = \frac{u}{R} n_{eff}$ where $u = nd$. This means the curved waveguide induce an effective DC field $f\left(n\right)=\Delta n_{eff}\cdot k_0 = Fn$ for n^{th} waveguide where $F = \frac{d}{R}k_0 n_{eff} = \frac{2\pi n_{eff} d}{R\lambda}$.

Supplementary Note 2

The corresponding dynamic evolution of the bent Hermitian photonic lattice under equivalent DC field can be expressed as:

$$\frac{d}{dz}A_n = i\kappa\left(A_{n-1} + A_{n+1}\right) + iFnA_n \tag{4}$$

where A_n is the mode envelop for n^{th} waveguide and κ is the coupling coefficient between adjacent waveguides.

Take a Fourier transformation A_n in Eqs (4),

$$A_n\left(z\right) = \frac{1}{\sqrt{2\pi}}\int_{-\pi}^{\pi}\tilde{A}\left(\rho,z\right)\exp\left(i\rho n\right)d\rho\,,\quad \tilde{A}\left(\rho,z\right) = \frac{1}{\sqrt{2\pi}}\sum_n A_n\left(z\right)\exp\left(-i\rho n\right)$$

we can get

$$\left(\frac{\partial}{\partial z} + F\frac{\partial}{\partial\rho} - i2\kappa\cos\rho\right)\tilde{A}\left(\rho,z\right) = 0 \tag{5}$$

The supermodes of the array can be represented as $A_n^m\left(z\right) = \Psi_m(n)e^{i\beta_{WSL}^m z}$, β_{WSL}^m is the wave number of the supermode m, $\Psi_m(n)$ is mode of the individual waveguide.

From Eqs (5), we can get $\tilde{\Psi}_m(\rho) = \exp\left(\frac{1}{F}\left(-i\beta_{WSL}^m\rho - 2i\kappa\sin\rho\right)\right)$, since $\tilde{\Psi}_m(\rho)$ is a periodic function which means $\tilde{\Psi}_m(-\pi) = \tilde{\Psi}_m(\pi)$, we can prove that Wannier-Stark wave numbers should satisfy that the condition $\beta_{WSL}^m = mF$, $m = 0, \pm1, \pm2, \cdots$ which means that the wave numbers are equidistant so it is named Wannier-Stark ladder while $\Psi_m(n)$ is the corresponding Wannier-Stark eigenstates in the Wannier representation[2,3].

Supplementary Note 3

For the non-Hermitian photonic lattice, in the presence of this DC field the spectra have two interleaved Wannier-Stark ladders $\beta'^{m}_{\pm} = 2mF + \frac{i\gamma}{2} \pm i\varepsilon\left(\frac{\kappa}{F}, \frac{\gamma}{F}\right)$, $m = 0, \pm1, \pm2, \cdots$, where $\varepsilon\left(\frac{\kappa}{F}, \frac{\gamma}{F}\right)$ is a complex function of $\frac{\kappa}{F}$ and $\frac{\gamma}{F}$, which can be numerically solved through the propagator S for the differential system[4-6] $\frac{dx_{1,2}}{d\varphi} = \pm i\frac{\kappa}{F}\cos\left(\varphi\right)x_{1,2} + \frac{\gamma}{4F}x_{2,1}$ from $\varphi = 0$ to $\varphi = \pi$. Generally there are two complex valued WS ladders for the curved non-Hermitian lattice. However, for some critical parameters $\left(\frac{\kappa_c}{F_c}, \frac{\gamma_c}{F_c}\right)$, the spectrum can also be given by only one single WS ladder which means the second emission will disappear. For example, at a low γ limit, the second emission will disappear when $\frac{\kappa}{F}$ satisfies $J_0\left(\frac{4\kappa}{F}\right) = 0$. So at some critical bending radii, e.g. $R_c = 32.7\mu m$, the second emission can be almost eliminated as shown in Supplementary Figure 1. The center radius R chosen for our experiment is away from this critical value and in turn the second emission can be observed and studied.

Supplementary Note 4

A tunable laser (Photonetics, 1,500-1,640 nm) source emitting light with a wavelength of 1,550 nm is coupled into a lensed tapered single mode fiber. The three-paddle polarization controller is used to align the state of polarization of the output light from the lensed fiber to be quasi-TE required for efficient coupling into the silicon waveguide on the SOI chip. The tip of the lensed tapered fiber is bonded onto a glass slide by using a low melting point wax, so is the SOI chip. In this way, when the glass slide is placed and fixed on the scanning translation stage of the SNOM (SNOM-100 Nanonics), the light coupling condition between the chip and the fiber can be maintained throughout the measurement. A metal-coated fiber tip with a 50nm radius is used as a probe to scatter and collect the near-field optical signal for analysis in a single-photon detector. The high-precision near-field electric field distribution is acquired by translating the chip under the probe.

Supplementary References

1. Lenz, G., Talanina, I. & de Sterke, C.M. Bloch oscillations in an array of curved optical waveguides. *Phys. Rev. Lett.* **83**, 963-966 (1999).
2. Peschel, U., Pertsch, T. & Lederer, F. Optical Bloch oscillations in waveguide arrays. *Opt. Lett*. **23**, 1701-1703 (1998).
3. Pertsch, T., Dannberg, P., Elflein, W., Brauer, A. & Lederer, F. Optical Bloch oscillations in temperature tuned waveguide arrays. *Phys. Rev. Lett.* **83**, 4752-4755 (1999).
4. Longhi, S. Bloch Oscillations in Complex Crystals with PT Symmetry. *Phys. Rev. Lett.* **103**, 123601 (2009).
5. Breid, B. M., Witthaut, D. & Korsch, H. J. Bloch–Zener oscillations. *New J. Phys.* **8**, 110 (2006).
6. Dreisow, F., Szameit, A., Heinrich, M., Pertsch, T., Nolte, S., Tünnermann, A. & Longhi, S. Bloch-Zener oscillations in binary superlattices. *Phys. Rev. Lett.* **102**, 076802 (2009).
